# Stunting and associated factors among under-five children in Wukro town, Tigray region, Ethiopia: a cross sectional study

**DOI:** 10.1186/s13104-019-4535-2

**Published:** 2019-08-14

**Authors:** Tesfay Tsegay Gebru, Yohannes Ashebir Tesfamichael, Muzayene Tilahun Bitow, Natnael Etsay Assefa, Gdiom Gebreheat Abady, Meresa Berwo Mengesha, Haftom Tesfay Gebremedhin

**Affiliations:** 1 Department of Nursing, Adigrat University, Adigrat, Ethiopia; 2 Department of Biomedical, Adigrat University, Adigrat, Ethiopia; 3 Department of Midwifery, Adigrat University, Adigrat, Ethiopia; 4 Department of Psychiatry, Adigrat University, Adigrat, Ethiopia

**Keywords:** Stunting, Factors, Wukro, Tigray region, Ethiopia

## Abstract

**Objective:**

The objective of the study was to assess the prevalence of stunting and associated factors among under-five children of Wukro town, Tigray, Ethiopia, 2017–2018.

**Result:**

Totally 394 under-five children were participated in this study with a response rate of 98.5%. A total of 222 (56.3%) of respondents were females and 106 (26.95%) were in the age group of 12–23 month. One hundred ninety-eight (50.3%) of the participants were between 2 and 3 in birth order and 194 (49.2%) had 4 to 5 house hold size. The overall prevalence of stunting was 194 (49.2%). Being female and presence of washing facilities nearby latrine were significantly associated with stunting. Under-five female children were 35.4% lower odds of stunting compared to male children (p = .041, OR = .644, and 95% CI (.422, .983)).

**Electronic supplementary material:**

The online version of this article (10.1186/s13104-019-4535-2) contains supplementary material, which is available to authorized users.

## Introduction

Under-nutrition causes 175 deaths per 1000 children in low-income compared to high-income countries. Malnutrition affects all individuals, but young children are the most vulnerable because of their high nutritional requirements for growth and development [1, 2]. Globally in 2011, 165 million children were stunted and these burdens were not distributed evenly worldwide [3]. Children who suffered from chronic malnutrition were more likely to achieve lower educational levels than healthy children [4]. Numbers of children stunted were declined from 255 million in 1999 to 159 million in 2014 [5].

Stunting and other forms of under nutrition are clearly a major contributing factor to child mortality, disease, and disability. A severely stunted child faces four times higher risk of dying, poor school achievement and poor school performance [3]. In Ethiopia, 40% and 19% of under-five children were moderately, and severely stunted respectively [6]. Similarly 16% of repetitions in school were because of chronic malnutrition and Ethiopia costs 55.5 billion-ETB for under nutrition prevention and management which accounts for 16.5% of the country’s GDP. The country had an estimated 378,591 child mortality related with under nutrition, from 2004 to 2009 [4]. Therefore the aim of this study was to assess the prevalence of stunting and associated factors among under-five children of Wukro town, Tigray region, Ethiopia.

## Main text

### Methods

#### Study design, period and participants

A community-based cross-sectional study design was conducted among under-five children from December 2017 to January 2018.

#### Sample size and sampling procedure

A single population proportion formula was employed to estimate the sample size with a consideration of

- p = 45.7% stunting (Ethiopian EDHS 2014)
- Z = standard normal distribution curve value for the 95% confidence interval (1.96)
- d = the margin of error or accepted error = 5% (.05)

5% non-response and the final sample size for this study were 400 participants.

Wukro town has three kebeles, out of this kebele 01 and 03 were included in the study randomly through the lottery method. Proportional allocation of subjects to each kebele was employed based on the number of under-five children and finally the study participants were selected through a systematic method based on the arrangement of houses. For households with more than one child, one child was selected randomly.

#### Data collection instruments and procedure

A structured questionnaire was developed by the principal investigator after reviewing different related literatures with required modification based on outcome variables and their predictors. The questionnaire was prepared first in English then translated to the local language (Tigrigna). To check the consistency of the translation; retranslation to English was done by another translator.

The questionnaire includes socio-demographic characteristics of care giver/family and child, child health condition, maternal health care, environmental health and anthropometric measurements.

The questionnaire was pre-tested on 5% of the same source population other than sampled population. Based on the pre-test, questions were revised, and edited. Finally, Tigrigna version questionnaire was used for data collection.

#### Variables

Dependent variable: under-five stunting.

Independent variable: socio-demographic variables, child caring practices, maternal characteristics and environmental Health condition.

#### Definition of terms

Stunting: Height-for-age below − 2 SD of median of the standard curve and severe stunting below − 3 SD [7].

Height/length measurement: Body length of children up to 23 months age was measured without shoes and the heights were read to the nearest 0.1 cm by using a horizontal wooden length board with movable headpiece and the infant in a recumbent position. However, the height of children 24 months and above was measured using a vertical wooden height board by placing the child on the measuring board, and child standing upright in the middle of board. The child’s head, shoulders, buttocks, knees and heels touching the board.

#### Data processing and analysis

Anthropometric result was entered into ENA to calculate Z-score. The collected data and result of Z-score were entered into SPSS version 20.0. Multi-collinearity was assessed through VIF and was found satisfied. Variables with a p-value less than .25 on bi-variate analysis were entered into the multivariable analysis. On multi-variant logistic regression analysis adjusted odds ratio with its 95% confidence interval was used to ascertain the association between dependent and independent variables. The level of significance was taken at α < .05.

### Results

#### Socio-demographic characteristics

Totally 394 under-five children with a response rate of 98.5% were participated in the study. Out of this 222 (56.3%) of participants were under-five female children. Orthodox christianity was the dominant religion consisting of 341 (86.5%) and 360 (91.4%) of the head of households were father. Almost all children were delivered at health facilities and cared by their mother but 104 (26.4%) decided on the use of money by mother and father jointly (Additional file 1: Table S1).

The main reason to stop taking breast feeding of under-five children was 167 (78%) because of age, and 392 (99.5%) of children was immunized according to their age. Among the children, 89 (22.6%) were experienced diarrhea in the last 2 weeks (Table 1).

**Table 1:** Health care practice and morbidity of under-five children in Wukro town, Tigray, North Ethiopia, 2017–2018 (N = 394)

Variable		Frequency	Percent (%)
Child ever taken to health facility	Yes	144	36.5%
	No	250	63.5%
Diarrhea in the last 2 week	Yes	89	22.6%
	No	305	77.4
ARI in the last 2 week	Yes	55	14.0
	No	339	86.0
Fever in the last 2 week	Yes	29	7.4
	No	365	92.6
Measles in the last 2 week	Yes	8	2.0
	No	386	98.0
Time of initiation of Breast feeding	Within one hour	376	95.4
	1-24 hour	17	4.3
	After 24 hour	1	.3
Child still taking breast feeding	Yes	181	45.9
	No	213	54.1
Duration of breast feeding	<24 month	108	50.7
	>=24 month	105	49.3
Initiation of complementary feeding	Before six month	8	2.0
	At 6 month	334	84.8
	>6 month	19	4.8
	Not introduced still now	33	8.4
Materials used for complementary feeding	Cup	148	41.0
	Bottle	69	19.1
	Spoon	143	39.6
	Hand	1	0.3
Vitamin A received in the last 6 month	Yes	310	78.7
	No	84	21.3
Preceding birth interval of baby	<=1 year	390	99.0
	1-2 year	4	1.0
Number of under five children	1	317	80.5
	>=2	77	19.5

Seventy-six (19.3%) of mothers gave their first birth before 18 years, in addition 283 (71.8%) and 368 (93.4%) of mothers were taken extra food during pregnancy and lactation respectively. A total of 388 (98.5%) of mothers had ANC follow up and 303 (76.9%) used FP but Depo-Provera were used by 230 (75.9%) mothers.

In all of the households, the sources of drinking water were tap water, and all households had nearby water source and latrine (Table 2).

**Table 2:** Hygienic condition of house hold of under-five children in Wukro town, Tigray, North Ethiopia, 2017–2018, (N = 394)

Variable		Frequency	Percent (%)
Type of latrine	Private pit/wooden slab	115	29.2
	Private pit/cement slab	279	70.8
Presence of washing facilities nearby latrine	Yes	258	65.5
	No	136	34.5
Use of soap for hand washing after toileting	Yes	313	79.4
	No	81	20.6
Type of waste disposal method	Open field	1	.3
	Common pit	393	99.7
Care giver wash hand before preparing food	Yes	392	99.5
	No	2	.5
Floor of the house	Earth floor	59	15
	Ceramics floor	335	85.0

#### Prevalence of under-five stunting

In this study the overall prevalence of stunting was 194 (49.2%).

#### Factors associated with under-five stunting

Before multivariate analysis, multi-collinearity diagnosis was assessed and was not found. On multivariate analysis sex of the child and presence of washing facilities nearby latrine were significantly associated with stunting (Table 3).

**Table 3:** Factors associated with stunting in Wukro town, North Ethiopia, 2017/18

Variable	Nutritional status		COR (95% CI)	AOR (95% CI)	P-Value
	Normal, n (%)	Stunted, n (%)			
Sex of the child					
Male	74 (43%)	98 (57%)	1	1	
Female	126 (56.8%)	96 (43.2%)	.575 (.385, .860)	.644 (.422, .983)	.041*
Mother’s marital status					
Single	5 (31.3%)	11 (68.7%)	1	1	
Married	189 (51.5%)	178 (48.5%)	.428 (.146, 1.257)	.378 (.057, 2.529)	.316
Divorced	6 (54.5%)	5 (45.5%)	.379 (.077, 1.856)	.394 (.070, 2.207)	.290
Mother’s Occupation					
House wife	154 (50%)	154 (50%)	1	1	
Merchant	28 (49.1%)	29 (50.9%)	1.036 (.588, 1.823)	.699 (.354, 1.383)	.304
Government employed	18 (62.1%)	11 (37.9%)	.611 (.279, 1.337)	.595 (.239, 1.481)	.264
Monthly Income of family	200 (50.8%)	194 (49.2%)	1.000 (1.00, 1.00)	1.000 (1.00, 1.00)	.853
Gestational Age of the child	200 (50.8%)	194 (49.2%)	.875 (.722, 1.060)	.957 (.772, 1.187)	.691
Birth weight of the baby	200 (50.8%)	194 (49.2%)	1.000 (.999, 1.00)	1.000 (.999, 1.000)	.009
Presence of fever in the last two weeks					
Yes	18 (62.1%)	11 (37.9%)	1	1	
No	182 (49.9%)	183 (50.1%)	1.645 (.756, 3.58)	1.797 (.760, 4.246)	.182
Mother take extra food during lactation					
Yes	183 (49.7%)	185 (50.3%)	1	1	
No	17 (65.4%)	9 (34.6%)	.524 (.228, 1.205)	.476 (.196, 1.156)	.101
Presence of washing facilities nearby latrine					
Yes	146 (56.6%)	112 (43.4%)	1	1	
No	54 (39.7%)	82 (60.3%)	1.979 (1.297,3.021)	2.363 (1.320,4.229)	.004*

*variables which have significant association with stunting

### Discussion

Prevalence of under-five stunting was high in Wukro town. The study noted that sex of the child, absence of diarrhea in the last 2 week, and presence of washing facilities nearby latrine were significantly associated with under-five stunting. There is high prevalence of under-five stunting which could a possible alarm the government on children failure to grow physically and mentally that result on poor productivity and school performance during adolescent and adulthood time.

According to this study 376 (95.4%) of under five children were initiated breast feeding within 1 h of delivery, which is comparably higher than study done in Oromia Region [8]. The difference might be due to dissemination of information on the advantage of early initiation breast feeding through Medias and health care workers and the possible reason for this is due time difference.

The result of this study indicated that 96 (24.3%) and 98 (24.9%) of under-five children were stunted and severely stunted respectively. This is consistent with the study conducted in Shire Endassilasie, Tigray [9] but comparatively lower than miniEDHS, 2014, [6]. The discrepancy might be due to small sample size compared to that of national data of mini EDHS, 2014.

According to this study female under-five children were lower odds (35.4%) of stunting compared male. This is consistent with the study conducted in Somali region [10]. Most of the studies in Ethiopia indicated that male children are more stunted than their counterparties.

The prevalence of stunting in the study area was 49.2% which was almost comparable with studies conducted in Amhara region (51.1%) [11], Sidama zone (50.3%) [12], and Oromia Regional State (47.6%), [8] but relatively higher than the national figure (38%) [13], and other parts of the country, like 26.6% in southern region [14] and 24.9% in Northwest Ethiopia [15]. However, it was lower than the finding from Southeast Amhara region (60.6%) [16].

### Conclusion and recommendations

Based on the finding of this research significant numbers of mothers were not taken extra food during pregnancy and lactation. There was high prevalence of stunting of under-five children. Being male, and presence of washing facilities nearby latrine were associated with increased risk of stunting. Therefore, special emphasis should also be provided feeding of pregnant and lactating mothers and provision of washing facilities nearby latrine to each household is required. Further research on impact of malnutrition should be recommended.

## Limitation of the study

- The nature of study design could not show seasonal variation and temporal relationship of cause and effect of cause and outcome.
- There is potential recall bias among respondents answering questions relating to events happening in the past like as history of diarrhea.

## Additional file

**Additional file 1: Table S1.** Socio-demographic characteristics of households of under five children in Wukro town, eastern zone, Tigray regional state, North Ethiopia, 2017–2018.

## Data Availability

The datasets during and/or analyzed during the current study available from the corresponding author on reasonable request.

## Abbreviations

AIDS: acquired immune deficiency syndrome
ANC: antenatal care
ARI: acute respiratory infections
BMI: body mass index
BSC: Bachelors of Science
EDHS: Ethiopian Demographic Health survey
ENA: Emergency Nutritional Assessments
ETB: Ethiopian Birr
FP: family planning
GDP: gross domestic product
HIV: human immunodeficiency virus
LBW: low birth weight
MSC: Master of Science
SD: standard deviation
SPSS: Statistical package for social science
VIF: variance inflation factor
