# Supplementary material for: Stunting and associated factors among under-five children in Wukro town, Tigray region, Ethiopia: a cross sectional study
Source: BMC Res Notes. 2019 Aug 14;12:504. doi: 10.1186/s13104-019-4535-2 (PMC6693130; doi:10.1186/s13104-019-4535-2)
Supplement: Supplementary file 1 — Additional file 1: Table S1. Socio-demographic characteristics of households of under five children in Wukro town, eastern zone, Tigray regional state, North Ethiopia, 2017–2018. [file 13104_2019_4535_MOESM1_ESM.docx]

**Additional File 1: Socio-demographic characteristics of households of under five children in Wukro town, eastern zone, Tigray regional state, North Ethiopia, 2017/18.**

| Variable | | Frequency (N=394) | Percent (%) |
| --- | --- | --- | --- |
| Age of the child in month | 0-6 | 37 | 9.4 |
|  | 6-11 | 55 | 14.0 |
|  | 12-23 | 106 | **26.9** |
|  | 24-35 | 85 | 21.6 |
|  | 36-47 | 77 | 19.5 |
|  | 48-59 | 34 | **8.6** |
| Sex of the child | Male | 172 | 43.7 |
|  | Female | 222 | 56.3 |
| Birth Order of the child | 1^st^ | 138 | 35.0 |
|  | 2-3 | 198 | **50.3** |
|  | >=4 | 58 | 14.7 |
| Household size | 1 | 1 | .3 |
|  | 2-3 | 127 | 32.2 |
|  | 4-5 | 194 | 49.2 |
|  | 6+ | 72 | 18.3 |
| Religion | Orthodox | 341 | 86.5 |
|  | Muslim | 53 | 13.5 |
| Mothers Education | Illiterate | 46 | 11.7 |
|  | Primary | 142 | 36.0 |
|  | Secondary and above | 206 | 52.3 |
| Marital status of mother | Single | 16 | 4.1 |
|  | Married | 367 | 93.1 |
|  | Divorced | 11 | 2.8 |
| Fathers education | Illiterate | 27 | 6.9 |
|  | Primary | 124 | 31.5 |
|  | Secondary and above | 243 | 61.6 |
| Mothers occupation | house wife | 308 | 78.1 |
|  | Merchant | 57 | 14.5 |
|  | government employed | 29 | 7.4 |
| Monthly income of family in Ethiopian Birr | <750 | 1 | .3 |
|  | 750-1500 | 38 | 9.6 |
|  | >1500 | 355 | 90.1 |
| Ethnicity of the family | Tigray | 391 | 99.2 |
|  | Amhara | 3 | .8 |
